# Radiotherapy and Smell Function in Head and Neck Cancer: A Nonrandomized Clinical Trial

**DOI:** 10.1001/jamanetworkopen.2025.48547

**Published:** 2025-12-18

**Authors:** Geng-He Chang, Yao-Te Tsai, Meng-Hung Lin, Ming-Shao Tsai, Ethan I. Huang, Cheng-Ming Hsu, Chang-Hsien Lu, Miao-Fen Chen, Wen-Cheng Chen

**Affiliations:** 1 Department of Otolaryngology–Head and Neck Surgery, Chang Gung Memorial Hospital, Chiayi, Taiwan, Republic of China; 2 Health Information and Epidemiology Laboratory, Chang Gung Memorial Hospital, Chiayi, Taiwan, Republic of China; 3 Department of Medical Oncology, Chang Gung Memorial Hospital, Chiayi, Taiwan, Republic of China; 4 Department of Radiation Oncology, Chang Gung Memorial Hospital at Linkou, Taoyuan, Taiwan, Republic of China; 5 College of Medicine, Chang Gung University, Tao-yuan, Taiwan, Republic of China; 6 Graduate Institute of Clinical Medicine, College of Medicine, Chang Gung University, Taoyuan, Taiwan, Republic of China; 7 Department of Radiation Oncology, Chang Gung Memorial Hospital, Chiayi, Taiwan, Republic of China

## Abstract

**Importance:**

A substantial proportion of patients with head and neck cancer (HNC) experience smell dysfunction during or after radiotherapy (RT). However, prospectively collected data for smell dysfunction have been limited, especially in the era of intensity-modulated RT (IMRT).

**Objective:**

To evaluate the association of RT with olfactory function in patients with HNC receiving IMRT, including changes in olfactory function over time.

**Design, Setting, and Participants:**

This nonrandomized clinical trial enrolled patients with histologically confirmed HNC between January 1, 2021, and December 1, 2023, who underwent curative treatment with IMRT at Chang Gung Memorial Hospital in Taiwan. Participants scheduled for RT underwent olfactory function assessment using the Taiwan Smell Identification Test at multiple time points, including before RT; at the end of RT; and at 1, 3, 6, and 12 months after treatment. Nasal endoscopy was used to rule out confounding nasal conditions.

**Exposure:**

Intensity-modulated RT with and without concurrent chemotherapy.

**Main Outcomes and Measures:**

The association between Taiwan Smell Identification Test scores and olfactory region radiation dose was analyzed using linear regression, and an estimative model was developed to calculate a threshold dose.

**Results:**

A total of 63 patients (median [range] age, 55 [32-75] years; 52 male [82.5%]) with normal olfactory function before RT were included. The most common primary cancer site was the oral cavity and oropharynx (37 participants [58.7%]), followed by the nasopharynx (16 participants [25.4%]). A moderate positive correlation was observed between radiation dose and olfactory dysfunction. Receiver operating characteristic analysis identified 22 Gy as a potential inflection point for estimating olfactory impairment. By multivariable analysis, a mean radiation dose to the olfactory region exceeding 22 Gy was the only independent risk factor for olfactory dysfunction (odds ratio, 20.65; 95% CI, 2.60-164.35). Some patients treated with high-dose radiation experienced persistent olfactory dysfunction of up to 1 year.

**Conclusions and Relevance:**

This study found a dose-dependent association between RT and olfactory dysfunction in patients with HNC, identifying 22 Gy as an important inflection point. To minimize the risk of long-term olfactory impairment, optimizing RT planning by carefully adjusting radiation dose and target area may be beneficial.

**Trial Registration:**

ISRCTN14947812

## Key Points

**Question:**

Is intensity-modulated radiotherapy associated with smell function in patients with head and neck cancer over time?

**Findings:**

In this nonrandomized clinical trial including 63 patients with a normal pretreatment sense of smell, higher radiation doses to the olfactory region were associated with moderate smell dysfunction. An inflection point dose of 22 Gy was identified; doses above this point increased the odds of dysfunction by 20-fold, and some patients had lasting impairment for up to 1 year.

**Meaning:**

These findings suggest that olfactory dysfunction is associated with the dose delivered to the olfactory region during intensity-modulated radiotherapy; therefore, limiting radiation exposure may reduce the risk of smell dysfunction in patients with head and neck cancer.

## Teaser

This nonrandomized clinical trial evaluates the association of intensity-modulated radiotherapy with olfactory function over time in patients with head and neck cancer in Taiwan.

## Introduction

Head and neck cancer (HNC) is the seventh most common cancer worldwide, with approximately 890 000 new cases and 450 000 deaths annually. Its increasing incidence highlights ongoing challenges in long-term survival.^1^ Radiotherapy (RT) is a key treatment modality that enhances tumor control and survival,^2^ especially with advances such as intensity-modulated RT (IMRT) and intensity-modulated proton therapy, which improve targeting and reduce damage to healthy tissues.^3^ However, RT-associated adverse effects, notably long-term olfactory and gustatory impairments,^4^ substantially impact patient quality of life^5^ by affecting taste, nutrition, and psychological well-being and potentially causing weight loss.^6,7^

The olfactory system’s anatomy^8^ makes it particularly vulnerable to radiation, especially in tumors near the nasal and paranasal regions where olfactory receptors are located in a mucosal area high in the nasal cavity. Damage to the olfactory bulb and epithelium can impair smell perception.^7,9^ Prior studies have suggested that RT may cause olfactory dysfunction; however, these studies were mostly retrospective or cross-sectional, limiting understanding of dynamic changes over time.^7,9,10^ In addition, concomitant nasal conditions, such as sinusitis or mucosal edema, were not well controlled, potentially confounding subsequent olfactory assessments.^11^

Although research has examined the association between radiation dose and olfactory impairment,^10^ the dose threshold for dysfunction remains undefined, hindering efforts to optimize RT plans for olfactory preservation. To address this limitation in knowledge, our prospective study investigated the association between radiation dose to the olfactory region and subsequent dysfunction, aiming to identify critical dose thresholds that elevate risk. The study also assessed how olfactory function evolves after treatment, offering insights into both immediate and long-term impacts, with the goal of improving patient outcomes through tailored RT strategies

## Methods

### Patient Enrollment and Study Process

In this nonrandomized clinical trial, we initially prospectively enrolled patients with histologically confirmed HNC who underwent curative (definitive or adjuvant) treatment with IMRT at Chang Gung Memorial Hospital between January 1, 2021, and December 1, 2023. This study was conducted in accordance with the ethical principles outlined in the Declaration of Helsinki^12^ and received approval from the Chang Gung Memorial Hospital Institutional Review Board. All participants were provided with comprehensive information regarding the study’s objectives and procedures, and written informed consent was obtained from each participant prior to enrollment. This study followed the Transparent Reporting of Evaluations With Nonrandomized Designs (TREND) reporting guideline. The study protocol is provided in Supplement 1.

To ensure accurate evaluation of olfactory function, inclusion criteria required patients to have baseline Taiwan Smell Identification Test (TWSIT) scores of 40 or higher, indicating normal olfaction. Exclusion criteria included a concomitant diagnosis of severe nasal septal deviation with hypertrophic turbinate, chronic rhinosinusitis with or without nasal polyps, head trauma, a history of RT to the head and neck region, or baseline TWSIT scores less than 40 (hyposmia and anosmia).

### Olfactory Function Assessment

Olfactory function was evaluated using the TWSIT, a validated tool designed to accurately assess olfactory function in Taiwanese people.^13^ The test comprises 8 familiar odorants (honey peach, passion fruit, cantaloupe, lemon, smoked plum, garlic, coffee, and jasmine) selected for their high identification rate (>95%) among Taiwanese individuals, ensuring cultural relevance and diagnostic accuracy. Each odor was tested twice, and scores were assigned based on accuracy and confidence, as follows: correct responses, 3 points; partially confident responses, 1 to 2 points; and incorrect responses, 0 points. The total score ranged from 0 to 48, with olfactory function classified as follows: for individuals aged 56 years or older, normosmia was defined as a score of 40 to 48, hyposmia as 12 to 39, and anosmia as 0 to 11; for those aged 55 years or younger, normosmia was defined as a score of 44 to 48, hyposmia as 12 to 43, and anosmia as 0 to 11. Given that the study population had a relatively high median age (approximately 56 years), a TWSIT score threshold of 40 was used to define normosmia. To evaluate olfactory function over time in patients undergoing RT for HNC, assessments were conducted at 6 specific time points: before the initiation of RT; at the end of RT; and at 1, 3, 6, and 12 months after treatment. Before each assessment, a nasal endoscopic examination was performed to ensure that the patient’s nasal condition was suitable for testing. If a condition such as sinusitis or an upper respiratory tract infection was detected, olfactory testing was postponed until the condition resolved. No olfactory function test was done if the patient had fully recovered from previous smell dysfunction after RT.

### Measurement of RT Dose to the Olfactory Region

The olfactory fossae, located in the anterior cranial fossa and containing the olfactory bulb of the olfactory nerve, are contoured in axial, coronal, and sagittal planes during IMRT treatment planning (eFigure 1A-C in Supplement 2).^14^ The fossa floor is formed by the medial lamella of the cribriform plate, bordered laterally by the lateral lamella and medially by the perpendicular plate.^14^ The anterior boundary is defined at the junction of the posterior wall of the frontal sinus and the cribriform plate, while the posterior boundary lies at the junction of the posterior edge of the cribriform plate and the anterior edge of the planum sphenoidale.^14^ Radiotherapy dose distributions were then calculated to determine the mean and maximum doses delivered to the defined olfactory region. These precise delineations enabled an accurate assessment of dose-response relationships in subsequent analyses.

### Statistical Analysis

The study analyzed the association between radiation dose to the olfactory region and TWSIT score reduction using linear regression, assessing the strength of correlations using the *R*^2^ value. Receiver operating characteristic (ROC) curve analysis identified the optimal radiation dose for estimating olfactory dysfunction, defined as a post-RT TWSIT score of less than 40, with Youden index used to calculate the cutoff and the area under the curve (AUC) to evaluate estimative accuracy.

To assess the probability of olfactory dysfunction across a range of radiation doses, a logistic regression model was constructed to generate a dose-response probability curve. We used SAS, version 9.4 (SAS Institute Inc) to fit the model using the proc logistic procedure, treating the radiation dose as the independent variable. We then used an effect plot to visualize the association. The plot illustrates how the estimated probability of olfactory dysfunction changes with varying doses. Additionally, multivariable logistic regression analysis was conducted to adjust for potential confounders, including age, sex, and clinical characteristics, and to estimate the odds ratio of olfactory dysfunction in patients receiving radiation doses above the identified threshold compared with those receiving radiation doses below the threshold. A 2-sided *P* < .05 was considered statistically significant. All data analyses were performed using SAS, version 9.4.

## Results

### Baseline Characteristics

**Figure 1.**
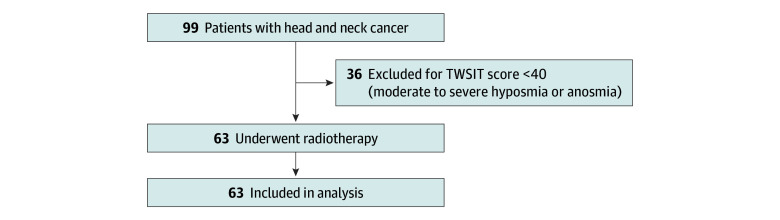
Participant Recruitment and Analysis Flow Diagram TWSIT indicates Taiwan Smell Identification Test.

Of 99 prospectively enrolled patients with histologically confirmed HNC who underwent curative IMRT, 63 (median [range] age, 55 [32-75] years; 11 female [17.5%] and 52 male [82.5%]) met the study inclusion criteria and were enrolled in the formal analysis (Figure 1). The Hosmer-Lemeshow test showed no evidence of poor fit (*P* = .15) (eFigure 2 in Supplement 2). The demographic and clinical characteristics of the study population are summarized in Table 1. A total of 37 participants (58.7%) had an education level above junior high school, and 32 (50.5%) reported a smoking history of less than 10 pack-years, while 31 (49.2%) reported a smoking history of 10 pack-years or more. All participants undergoing continued testing had ceased smoking. The most common primary cancer site was the oral cavity and oropharynx (37 participants [58.7%]), followed by the nasopharynx (16 participants [25.4%]). According to the TNM staging system, most patients had advanced-stage disease, with 13 (20.6%) with stage III, 19 (30.2%) with stage IVa, and 13 (20.6%) with stage IVb cancer. Regarding treatment, 40 participants (63.5%) underwent curative concurrent chemoradiotherapy, 14 (22.2%) received curative RT alone, and 9 (14.3%) received chemotherapy followed by concurrent chemoradiotherapy. Of all participants, 29 (46.0%) received adjuvant and 34 (54.0%) received definitive RT. The median total radiation dose was 68 Gy (range, 36-77 Gy), with a mean olfactory region dose of 3 Gy (range, 0-62 Gy).

**Table 1.** Characteristics of the Study Population

Characteristic	Participants, No. (%)		
	All (N = 63)	Olfactory dysfunction	
		No (n = 52)	Yes (n = 11)
Sex			
Female	11 (17.5)	8 (15.4)	3 (27.3)
Male	52 (82.5)	44 (84.6)	8 (72.7)
Age, median (range), y	55 (32-75)	57 (32-75)	55 (37-72)
Education			
Junior high school or less	26 (41.3)	23 (44.2)	3 (27.3)
Above junior high school	37 (58.7)	29 (55.8)	8 (72.7)
Performance status^a^			
0-1	59 (93.7)	48 (92.3)	11 (100)
2-3	4 (6.3)	4 (7.7)	0
Tobacco smoking history, pack-years			
<10	32 (50.8)	25 (48.1)	7 (63.6)
≥10	31 (49.2)	27 (51.9)	4 (36.4)
Primary cancer site			
Oral cavity and oropharynx	37 (58.7)	33 (63.5)	4 (36.4)
Nasopharynx	16 (25.4)	10 (19.2)	6 (54.5)
Hypopharyngeal	3 (4.8)	3 (5.8)	0
Laryngeal	3 (4.8)	3 (5.8)	0
Hard palate	1 (1.6)	1 (1.9)	0
Parotid	2 (3.1)	2 (3.8)	0
*NUTM1* midline carcinoma	1 (1.6)	0	1 (9.1)
T stage			
T1	12 (19.1)	9 (17.3)	3 (27.3)
T2	18 (28.6)	15 (28.8)	3 (27.3)
T3	12 (19.0)	10 (19.2)	2 (18.2)
T4a	14 (22.2)	11 (21.2)	3 (27.3)
T4b	7 (11.1)	7 (13.5)	0
N stage			
N0	20 (31.8)	17 (32.7)	3 (27.3)
N1	13 (20.6)	9 (17.3)	4 (36.4)
N2a	8 (12.7)	5 (9.6)	3 (27.3)
N2b	9 (14.3)	9 (17.3)	0
N2c	5 (7.9)	5 (9.6)	0
N3	8 (12.3)	7 (13.5)	1 (9.1)
Overall stage			
I	9 (14.3)	7 (13.5)	2 (18.2)
II	9 (14.3)	7 (13.5)	2 (18.2)
III	13 (20.6)	11 (21.1)	2 (18.2)
IVa	19 (30.2)	15 (28.8)	4 (36.4)
IVb	13 (20.6)	12 (23.1)	1 (9.1)
Treatment			
RT alone	14 (22.2)	13 (25.0)	1 (9.1)
CCRT	40 (63.5)	33 (63.5)	7 (63.6)
Chemotherapy and CCRT	9 (14.3)	6 (11.5)	3 (27.3)
Total radiation dose, median (range), Gy	68 (36-77)	66 (36-77)	70 (60-72)
Olfactory radiation dose, median (range), Gy	3 (0-62)	3 (0-56)	34 (0.8-62)

Abbreviations: CCRT, concurrent chemoradiotherapy; RT, radiotherapy.

^a^ Performance status 0 indicates fully active, able to perform all predisease performance without restriction; 1, restricted in physically strenuous activity but ambulatory and able to perform work of a light or sedentary nature (eg, light housework, office work); 2, ambulatory and capable of all self-care but unable to perform any work activities and is up and about more than 50% of waking hours; and 3, capable of only limited self-care and confined to bed or chair more than 50% of waking hours.

### Changes in Olfactory Function Over Time

**Figure 2.**
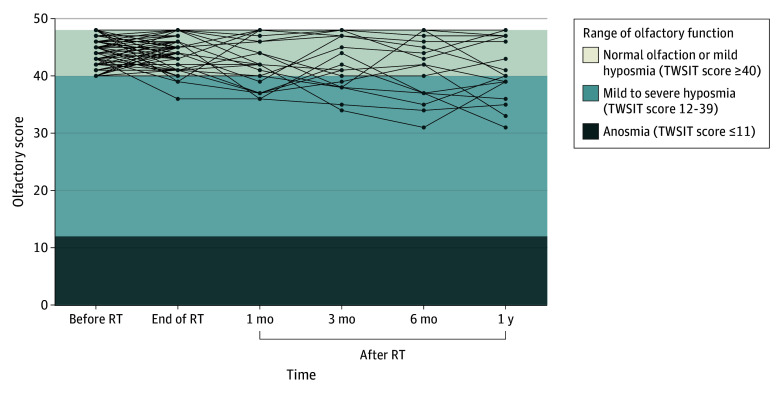
Longitudinal Changes in Olfactory Scores Over Time in Participants Undergoing Radiotherapy (RT) The lines represent the individual trends in olfactory changes based on Taiwan Smell Identification Test (TWSIT) scores for each of the 63 participants. Participants who received low radiation doses to the olfactory region and maintained normal olfactory scores (TWSIT score of >40 on a scale of 0-48) often discontinued follow-up assessments as their olfactory function did not deteriorate.

The longitudinal trends in olfactory scores for the 63 participants are illustrated in Figure 2, with assessments conducted at baseline (before RT); at the end of RT; and at 1, 3, 6, and 12 months after RT. Overall, olfactory scores declined after RT, followed by partial recovery in some patients during follow-up. Participants receiving low radiation doses to the olfactory region tended to maintain stable olfactory scores throughout the study period, with no significant changes observed. Two of 7 participants (28.6%) with olfactory dysfunction regained full olfactory function 2 to 3 years after RT.

### Correlation Between Radiation Dose and Reduction in Olfactory Scores

eFigure 3 in Supplement 2 illustrates the correlation between radiation dose to the olfactory region and the reduction in TWSIT scores. Linear regression analysis showed a moderate positive correlation; the regression slope of 0.128 suggested that for each 1-Gy increase in radiation dose, the TWSIT score decreased by a mean of 0.128 points (range, 0.083-0.173).

### ROC Curve Analysis for Estimating Olfactory Dysfunction

eFigure 4 in Supplement 2 presents the ROC curve used to evaluate the estimative accuracy of radiation dose in identifying patients at risk of olfactory dysfunction. Based on the Youden index, the optimal cutoff dose for estimating olfactory dysfunction was determined to be 22 Gy (AUC, 0.74; 95% CI, 0.52-0.96; sensitivity, 64%; specificity, 92%; *P* = .03). An interval validation of the ROC curve using bootstrap resampling (n = 1000) found an AUC of 0.74 (95% CI, 0.49-0.94). Based on the sensitivity of 64% and specificity of 92%, patients who receive an olfactory bulb radiation dose of less than 22 Gy may have a greater than 90% likelihood of not developing olfactory dysfunction. However, if the dose exceeds this value, approximately 64% may experience olfactory dysfunction.

### Estimative Model for Olfactory Dysfunction

**Figure 3.**
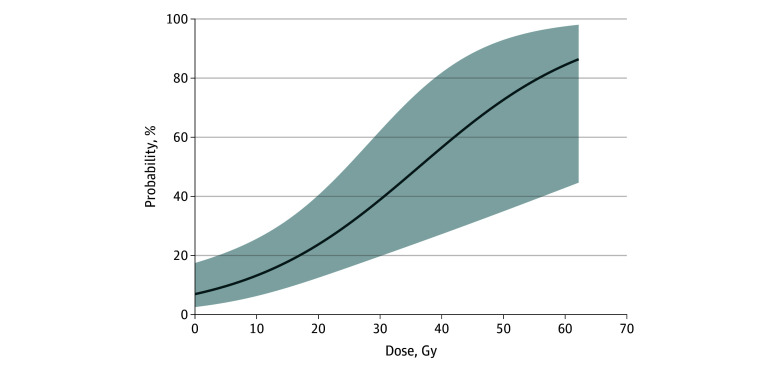
Estimated Probability of Olfactory Dysfunction Based on Radiotherapy Dose Shading indicates the 95% CI.

Figure 3 illustrates the estimative model for the probability of olfactory dysfunction (TWSIT score <40) based on radiation dose to the olfactory region. The model shows a dose-dependent increase in dysfunction probability, with a noticeable inflection point at approximately 22 Gy. The squared term of dose was reintroduced to examine the nonlinear effects. The results indicated that the squared term was not significant. Nonetheless, the nonlinear hypothesis was tested, and the nonsignificance of the squared term supported the initial linear assumption, suggesting that the association between dose and outcome could be effectively described by a linear model. At this dose level, the probability of dysfunction was approximately 25%, and beyond this infection point, the slope of the curve steepened, indicating a marked increase in risk. The multivariable analysis confirmed that a mean radiation dose to the olfactory region exceeding 22 Gy was an independent risk factor for olfactory dysfunction (odds ratio, 20.65; 95% CI, 2.60-164.35) (Table 2).

**Table 2.** Factors Associated With the Occurrence of Olfactory Dysfunction Within 1 Year After RT for Head and Neck Cancer

Variable	Participants with olfactory dysfunction, No. (%)^a^	OR (95% CI)	
		Crude	Adjusted^b^
Sex			
Female	3 (27.3)	2.06 (0.45-9.49)	0.30 (0.03-3.51)
Male	8 (15.4)	1 [Reference]	1 [Reference]
Age, y			
<55	4 (14.3)	1 [Reference]	1 [Reference]
≥55	7 (20.0)	1.50 (0.39-5.75)	2.36 (0.43-12.98)
Smoking history, pack-y			
<10	7 (21.9)	1 [Reference]	1 [Reference]
≥10	4 (12.9)	0.53 (0.14-2.03)	0.53 (0.09-3.17)
Stage			
Early (I-III)	6 (19.4)	1 [Reference]	1 [Reference]
Advanced (IV)	5 (15.6)	0.77 (0.21-2.85)	0.52 (0.09-3.08)
Treatment			
RT alone	1 (7.1)	1 [Reference]	1 [Reference]
CCRT	7 (17.5)	2.76 (0.31-24.65)	3.16 (0.23-43.03)
Chemotherapy and CCRT	3 (33.3)	6.50 (0.56-76.11)	4.59 (0.18-120.08)
Olfactory mean dose, Gy			
≤22	5 (9.4)	1 [Reference]	1 [Reference]
>22	6 (60.0)	14.40 (3.01-68.86)	20.65 (2.60-164.35)

Abbreviations: CCRT, concurrent chemoradiotherapy; OR, odds ratio; RT, radiotherapy.

^a^ Defined as a Taiwan Smell Identification Test score of less than 40 (scale of 0-48).

^b^ Adjusted for sex, age, smoking history, stage, treatment, and olfactory mean dose.

### Olfactory Function in Participants Receiving a High Radiation Dose

eFigure 5 in Supplement 2 depicts longitudinal changes in olfactory scores among 12 participants who received more than 22 Gy to the olfactory region. The mean (SD) TWSIT score declined from a baseline of 45.58 (2.75) before RT to 42.50 (3.99) at the end of RT, reflecting an early decline in olfactory function. At 1 month after treatment, the mean (SD) score partially recovered to 42.83 (4.76), but slightly declined to 42.00 (5.44) at 3 months. Scores remained relatively stable but continued to show a gradual downward trend, reaching a mean (SD) of 42.17 (6.03) at 6 months and 41.60 (6.33) at 1 year. Although a downward trend in olfactory scores was observed in this group of participants, the decrease was not significant compared with pretreatment scores.

## Discussion

This nonrandomized clinical trial found a dose-dependent association of RT with olfactory function in patients with HNC. An inflection point dose of 22 Gy was identified, beyond which the risk of olfactory dysfunction significantly increased. Logistic regression analysis adjusting for sex, age, smoking history, cancer stage, and treatment modality confirmed that participants receiving more than 22 Gy to the olfactory region had a 20-fold higher risk of olfactory dysfunction. The estimative model further showed a steep rise in dysfunction probability above this dose, with some participants experiencing persistent impairment up to 1 year after treatment. These findings emphasize the need to carefully assess the dose and extent of the olfactory region during RT planning to prevent long-term olfactory dysfunction.

Previous research has emphasized that the impact of RT on olfactory function is predominantly determined by the radiation dose and the specific anatomic areas targeted.^7,10^ While some research has suggested that additional factors, such as RT techniques, patient age, and concurrent taste dysfunction, may contribute to olfactory impairment,^15^ others did not find significant associations with age, sex, or chemotherapy.^7,10^ A key aspect of establishing causality in these studies is to explore the presence of a dose-dependent association.

Brämerson et al^7^ reported that patients receiving radiation doses exceeding 10 Gy to the olfactory region exhibited significant reductions in olfactory detection and identification capabilities.^7^ However, their study was not longitudinal in design, as olfactory function was assessed only prior to RT and between 12 and 35 months after treatment (mean, 20 months). Similarly, Jalali et al^10^ observed a decline in olfactory threshold scores at doses greater than 135 μCi (to convert to becquerels, multiply by 37 000), the threshold values reported are not directly comparable due to methodological differences and the use of an outdated treatment modality (Cobalt-60).^10^ Our study is the first prospective, longitudinal investigation to our knowledge that used modern RT techniques to find a dose-response relationship for olfactory function.

The potential for olfactory recovery following RT remains uncertain due to heterogeneity in RT techniques, radiation doses and fields, study designs, olfactory assessment methods, and timing of evaluations.^6^ Some studies have reported partial recovery of olfactory function between 3 and 6 months after treatment, although most patients experienced incomplete restoration.^16,17^ In contrast, other research has shown that the olfactory threshold continues to decline within 12 months after RT completion.^10^ Additionally, while olfactory identification may remain relatively stable during the first year after treatment, changes in the olfactory threshold have been observed to emerge beyond 12 months and may persist for up to 5 years.^6,18^ Notably, the recovery trajectories of olfactory discrimination and identification appear to differ, suggesting that RT may impair various aspects of olfactory function through distinct pathophysiologic mechanisms.^6^ In this study, a gradual decline in olfactory function was observed, most notably in participants who received doses greater than 22 Gy, with measurable, but mild hyposmia. The decrease in TWSIT scores was less than 5, suggesting that the severity of olfactory impairment was mild and less pronounced than taste disturbances frequently associated with RT.^19^

The mechanisms underlying RT-induced olfactory damage remain under investigation. The existing literature provides some evidence supporting future research directions. Radiotherapy can directly damage the respiratory mucosa and neural synapses in the olfactory region, contributing to both transport and sensory olfactory dysfunction.^20^ The exposed nasal mucosa may develop edema, congestion, or mucosal thickening, obstructing odor entry into the olfactory region and resulting in conductive hyposmia. Studies have reported that olfactory function decline is more pronounced with higher radiation doses or when RT is delivered closer to the olfactory structures, supporting the underlying pathophysiologic mechanisms.^7,10^ Additionally, RT may exacerbate preexisting chronic nasal conditions, further worsening olfactory function. The observed partial recovery in some cases may be attributed to the regenerative capacity of the olfactory epithelium itself. Unlike other sensory systems, the olfactory system has a unique ability for neurogenesis, with basal cells serving as progenitors for olfactory receptor neurons.^6,10^ This regenerative process may explain the partial recovery seen in patients with moderate radiation exposure, as observed in this and other studies. However, the association of higher radiation doses (>22 Gy) with increased persistent dysfunction suggests that the regenerative capacity may be overwhelmed by radiation-induced damage to both the olfactory epithelium and the olfactory bulb. Additionally, radiation-induced fibrosis and vascular damage may further hinder recovery. Fibrosis within the nasal mucosa or around the olfactory bulbs can compromise the structural integrity necessary for neural regeneration and olfactory signal transmission.^9^ Moreover, vascular damage may impair oxygen and nutrient delivery to the olfactory epithelium, further limiting the recovery process. However, some studies have suggested that while odor discrimination substantially declines within 2 to 6 weeks after the start of RT, odor identification and threshold remain unaffected, indicating that the olfactory epithelium may be relatively resistant to radiation-induced damage.^9^ Additionally, phantosmias, which are more frequently observed in younger individuals, during brain irradiation, and with proton therapy, may also result from the activation of brain regions not typically associated with the olfactory networks, indicating a high-level central neural dysfunction rather than a peripheral mechanism.^15^ These findings highlight the dose-dependent nature of olfactory recovery and the need for RT protocols that balance tumor control with the preservation of nontarget structures, warranting further studies to explore the underlying pathophysiologic mechanisms.

### Limitations

This study had several limitations. First, the small sample size may limit the generalizability of the findings to the broader patient population with HNC. Although the Hosmer-Lemeshow test showed no evidence of poor fit (*P* = .15) (eFigure 2 in Supplement 2), the small sample might increase the risk of overfitting. Nonetheless, it is the largest prospective longitudinal study of olfactory function to date compared with recent reviews.^21^ Second, despite using a prospective design and controlling for nasal conditions through regular endoscopy, unmeasured variables, such as genetic predisposition and environmental influences, may have contributed to the outcomes. Third, the regional specificity of the TWSIT may restrict its effectiveness in non-Asian populations due to cultural odor differences. In addition, the absence of a minimal clinically important difference for TWSIT may make minor score changes clinically nonsignificant. However, the use of validated categorical thresholds for normosmia, hyposmia, and anosmia, which strongly correlate with the Traditional Chinese Version of the University of Pennsylvania Smell Identification Test (*r* = 0.874), helps define dysfunction based on crossing an abnormality threshold (<40).^13^ Fourth, the 12-month follow-up may not capture late recovery; 2 of 7 of our participants with olfactory dysfunction regained full olfactory function 2 to 3 years after RT. Smoking during recovery might also influence results, although all participants undergoing continued testing had ceased smoking. Finally, although we identified an inflection point dose of 22 Gy, interindividual variability suggests that this threshold may not be universally applicable.

## Conclusions

This nonrandomized clinical trial found that olfactory function declines in a dose-dependent manner after RT for HNC, with higher radiation doses associated with greater impairment. An inflection point dose of 22 Gy was identified, beyond which the risk of olfactory dysfunction increased significantly, with long-term dysfunction persisting for up to 1 year after treatment. In patients with HNC, careful planning of the radiation exposure range and dose to the olfactory region is essential to enhance treatment efficacy while preserving olfactory function and maintaining quality of life.

## Notes

Supplement 1.
Trial Protocol

Supplement 2. **eFigure 1.** Dose Distribution to the Olfactory Regions During Radiotherapy **eFigure 2.** The Results of Hosmer-Lemeshow Goodness-of-Fit Test **eFigure 3.** Correlation Between Radiotherapy Dose and the Reduction in Olfactory Scores From Before Radiotherapy to Within 1 Year After Radiotherapy **eFigure 4.** Receiver Operating Characteristic Curve for Estimating Olfactory Dysfunction Within 1 Year After Radiotherapy Based on Radiotherapy Dose **eFigure 5.** Changes in Olfactory Scores Over Time in Patients Receiving High-Dose Radiotherapy

Supplement 3.
Data Sharing Statement
