# Supplementary material for: Radiotherapy and Smell Function in Head and Neck Cancer: A Nonrandomized Clinical Trial
Source: JAMA Netw Open. 2025 Dec 18;8(12):e2548547. doi: 10.1001/jamanetworkopen.2025.48547 (PMC12715645; doi:10.1001/jamanetworkopen.2025.48547)
Supplement: Supplement 1. — Trial Protocol [file jamanetwopen-e2548547-s001.pdf]

Chang Gung Research Project (CMRPG6L0041-3)

Title: Prospective Investigation of Olfactory Function in Patients with Head and Neck Cancer Receiving Intensity-Modulated Radiotherapy (IMRT)

P.I. – Wen-Cheng Chen, MD
Professor, Department of Radiation Oncology, Chang Gung Memorial Hospital, Chiayi

Co-PI. - Geng-He Chang MD, PhD
Associate Professor, Departments of Otolaryngology-Head and Neck Surgery, Chang Gung Memorial Hospital, Chiayi

Study period: 2021/01/01 ~ 2023/12/31

English Abstract:

Background and Purpose:

Radiotherapy (RT) is an important modality to treat head and neck (H & N) cancer. Only a few studies have reported that smell function deteriorated in H & N cancer patients after RT. But how long does the olfactory disorder appear after RT? What is the relationship with radiation dose and field? Could it be restored? If yes, how long is the recovery time? There is currently no research to fully explore those important issues. In addition, there are obvious differences in the familiarity of olfactory samples between different races. Westerners' olfactory assessment tools might not be suitable for accurate evaluations of the variations in olfaction of Taiwanese H & N cancer patients before and after treatments. Currently, an available olfactory assessment tool has been developed specifically for Taiwanese people. Therefore, in order to comprehensively evaluate the olfactory function of patients with H & N cancer patients after receiving intensity-modulated radiotherapy (IMRT), we initiated this prospective longitudinal study. This study will investigate the time, degree and prognosis of olfactory disorders in the acute and chronic phases after RT treatments.

Methods:

The research population of this study is newly diagnosed H & N cancer patients who receive curative IMRT at Chiayi Chang Gung Memorial Hospital during the study period. It is estimated that 100 patients will be enrolled in three years. Before RT, at the end of RT, one month, three months, six months and one year after RT, those patients will be tested by the Taiwan version of the olfactory assessment tool to evaluate their olfactory status. In addition, the Taiwan version of Sino-nasal Outcome Test-22 will be used to assess the subjective nasal symptoms of those H & N cancer patients. Nasal endoscopy will be performed to investigate and record the state of the nasal cavity of them, and we will collect those patients' nasal secretions for testing to analyze the changes in the nasal microenvironment.

Keywords：Loss of smell, Olfactory dysfunction, Hyposomnia, Anosmia, head and neck cancer, radiotherapy, IMRT

Please provide an overview of the anticipated impacts that the implementation of this project may have on various aspects, including social, economic, and academic development, in no more than 150 words.

Loss of smell can reduce the quality of life, sometimes cause danger, such as inability to smell harmful substances, and reduce the taste of food and cause nutritional disorders. Based on the findings of this study, we can comprehensively realize the effect and prognosis of RT on the olfactory function in H & N cancer patients. Then, on the basis of this research, we will have the opportunity to research and develop effective therapies for preventing, attenuating or treating the radiation-associated complication.

Backgrounds & Purpose:

Common radiotherapy complications

Radiotherapy (RT) is an important method to treat Head and Neck (H & N) cancer patients. The area of the head and neck receiving RT often leads to complications of varying degrees, such as xerostomia, dental problems, taste disorders, mucositis, etc.[1] These problems can cause malnutrition in patients and seriously affect the quality of life.

Radiation-associated olfactory dysfunction

The endings of the olfactory nerve are located on the olfactory epithelium and bulb where the junction of the nasal cavity and anterior skull base, which is generally called the cribriform plate.[2] It is close to the nasopharynx and easily falls into the scope of radiotherapy for treating H & N cancer. Although there were few studies discussing on the alternation in the sense of smell of patients with H & N cancer after RT, sporadic studies have reported that these patients would have olfactory dysfunction after treatment. Veyseller *et al* used Connecticut Chemosensory Clinical Research Center (CCCRC) test, a commonly used tool for accessing olfactory function, to evaluate the changes of smell in 24 Nasopharyngeal cancer (NPC) cases after RT. The mean CCCRC score was 5.5 ± 1.1 for those NPC patients and 6.4 ± 0.4 for a control group, which indicated a significant reduction in the olfactory function ($p = 0.003$).[3]

The perception of smell plays an important role in daily life, for example, it can avoid inhaling harmful substances, which would cause damage of the respiratory system. In addition, the sense of smell is also related to the perception of taste.[4] Dysfunction of smell would deteriorate radiation-induced taste disorders, which could reduce appetite and affect the nutritional status of NPC patients.[4]

Lack of the past researches

There were fewer studies investigating the effect of RT on the sense of smell in patients with H

& N cancer, compared with the complications of oral cavity, such as gustatory function and mucositis. Therefore, it lacks several important information about RT-associated dysosmia, such as how long does it take to develop smell impairments after radiation? How is the degree of impact related to the dose and field of radiotherapy? Could the olfactory dysfunction recover? and how long will it take to recover? Therefore, we conduct this prospective study to explore these important issues in radiation induced smell impairments.

Common tools for testing smell function

At present, the following tools are mainly used for olfactory detection in the world, including the University of Pennsylvania Smell Identification Test (UPSIT) [5] and the Connecticut Chemosensory Clinical Research Center identification test (CCCRC) [6] in the Unites States, and the Sniffin Sticks test in Europe [7].

Odor familiarity

Regarding the choice of an adequate tool for the odor identification test, the most important factor is whether the tested race is familiar with the types of smell used in the detection tool.[8] For example, a certain smell in an odor identification test is quite familiar to one race, but this smell may be unfamiliar to another race, and herein, the tool is not suitable for the latter.

For Orientals' olfactory testing, the Traditional Chinese version of UPSIT (TC-UPSIT) is currently commonly used [9,10], but the flavors used in the test are still based on things familiar to Westerners, such as the odors of pizza, motor oil, strawberry and chocolate, but these odors might not be familiar to Taiwanese people (or even Asians).

Specific olfactory assessment for Taiwanese – "TWSIT"

H & N cancer is a common cancer in Taiwan and compared with other countries in the world,

the prevalence of H & N cancer in Taiwan is relatively high. Therefore, it is suitable and feasible to collect enough H & N cancer cases in Taiwan to investigate the effect and prognosis of RT-associated olfactory dysfunction. However, as described in the previous paragraph, only by choosing an appropriate tool for smell evaluation can obtain the accurate outcomes. Therefore, a designed odor identification test based on the smells familiar to Taiwanese is the most suitable tool for studying the issue.

A brief and reliable smell identification test named the Taiwan Smell Identification Test (TWSIT) was developed and available currently. The test includes a novel questionnaire to improve the precision in measuring smell deficits and provides clinicians with a convenient, validated, measure of olfactory function in Taiwanese patients.

Effects of sinusitis on olfaction

Past studies have also pointed out that sinusitis itself could cause olfactory dysfunction. But the sinusitis-related smell impairment is probably the most treatable form of olfactory dysfunction. Most studies reported that the effects are usually transient and incomplete.[11]

Patients with H & N cancer, especially NPC, was found to be prone to develop sinusitis during and after radiotherapy. Su et al studied 155 NPC cases without sinusitis before RT and found the incidence rates of sinusitis at the end of RT and at 1, 3, 6, 9, 12, and 18 months after RT were 32.9, 43.2, 61.3, 68.4, 73.5, 69.7, and 61.3 %, respectively.[12] The occurrence of sinusitis increased rapidly within the first 3 months after IMRT, and the number of sinusitis cases peaked at 6-9 months after IMRT and showed a trend toward stabilization after 1 year.[12]

In the past studies on discussing the effect of RT on smell in NPC patients, they only excluded NPC cases with chronic sinusitis before enrolling process, but no research evaluated the effect of sinusitis developing during and after radiotherapy on confounding the assessments of radiotherapy-associated olfactory alternation.

Olfactory disorders and nasal microenvironment

The plasma levels of interferon-γ (IFN-γ), interleukin (IL)-1b, IL-6, monocyte chemoattractant protein (MCP-1), tumor necrosis factor (TNF)-α, IL-2 and IL-5 were reported to be significantly increased in NPC patients and dramatically decreased after treatment.[13] But few studies investigating the cytokines in the nasal secretion before and after NPC treatment.

Whether the changes of cytokines in the nasal microenvironment have correlation with the alternation of olfactory function, which is worthy of research and the understanding of the relationship might provide clues to develop methods for preventing, attenuating or treating the radiotherapy-associated olfactory dysfunction.

References

1. Yeh SA, Tang Y, Lui CC, Huang YJ, Huang EY. Treatment outcomes and late complications of 849 patients with nasopharyngeal carcinoma treated with radiotherapy alone. Int J Radiat Oncol Biol Phys. 2005;62(3):672-679.
2. Nagayama S, Homma R, Imamura F. Neuronal organization of olfactory bulb circuits. Front Neural Circuits. 2014;8:98.
3. Veyseller B, Ozucer B, Degirmenci N, et al. Olfactory bulb volume and olfactory function after radiotherapy in patients with nasopharyngeal cancer. Auris, nasus, larynx. 2014;41(5):436-440.
4. Lilja M, Markkanen-Leppanen M, Viitasalo S, et al. Olfactory and gustatory functions after free flap reconstruction and radiotherapy for oral and pharyngeal cancer: a prospective follow-up study. European archives of oto-rhino-laryngology : official journal of the European Federation of Oto-Rhino-Laryngological Societies (EUFOS) : affiliated with the German Society for Oto-Rhino-Laryngology - Head and Neck Surgery. 2018;275(4):959-966.

5. Doty RL, Shaman P, Kimmelman CP, Dann MS. University of Pennsylvania Smell Identification Test: a rapid quantitative olfactory function test for the clinic. The Laryngoscope. 1984;94(2 Pt 1):176-178.
6. Cain WS. Testing olfaction in a clinical setting. Ear, nose, & throat journal. 1989;68(4):316, 322-318.
7. Kobal G, Hummel T, Sekinger B, Barz S, Roscher S, Wolf S. "Sniffin' sticks": screening of olfactory performance. Rhinology. 1996;34(4):222-226.
8. Hong SM, Park IH, Kim KM, Shin JM, Lee HM. Relationship between the Korean Version of the Sniffin' Stick Test and the T&T Olfactometer in the Korean Population. Clinical and experimental otorhinolaryngology. 2011;4(4):184-187.
9. Jiang RS, Su MC, Liang KL, Shiao JY, Wu SH, Hsin CH. A pilot study of a traditional Chinese version of the University of Pennsylvania Smell Identification Test for application in Taiwan. Am J Rhinol Allergy. 2010;24(1):45-50.
10. Yu CY, Wu RM. Application of the University Of Pennsylvania Smell Identification Test (traditional Chinese version) for detecting olfactory deficits in early Parkinson's disease in a Taiwanese cohort. J Parkinsons Dis. 2014;4(2):175-180.
11. Raviv JR, Kern RC. Chronic sinusitis and olfactory dysfunction. Otolaryngol Clin North Am. 2004;37(6):1143-1157, v-vi.
12. Su YX, Liu LP, Li L, et al. Factors influencing the incidence of sinusitis in nasopharyngeal carcinoma patients after intensity-modulated radiation therapy. European archives of oto-rhino-laryngology : official journal of the European Federation of Oto-Rhino-Laryngological Societies (EUFOS) : affiliated with the German Society for Oto-Rhino-Laryngology - Head and Neck Surgery. 2014;271(12):3195-3201.
13. Jin YB, Zhang GY, Lin KR, et al. Changes of plasma cytokines and chemokines expression level in nasopharyngeal carcinoma patients after treatment with definitive intensity-modulated

radiotherapy (IMRT). PloS one. 2017;12(2):e0172264.

14. Thamboo A, Santos RC, Naidoo L, Rahmanian R, Chilvers MA, Chadha NK. Use of the SNOT-22 and UPSIT to appropriately select pediatric patients with cystic fibrosis who should be referred to an otolaryngologist: cross-sectional study. JAMA Otolaryngol Head Neck Surg. 2014;140(10):934-939.

15. Toma S, Hopkins C. Stratification of SNOT-22 scores into mild, moderate or severe and relationship with other subjective instruments. Rhinology. 2016;54(2):129-133.

16. Psaltis AJ, Li G, Vaezeafshar R, Cho KS, Hwang PH. Modification of the Lund-Kennedy endoscopic scoring system improves its reliability and correlation with patient-reported outcome measures. The Laryngoscope. 2014;124(10):2216-2223.

Materials & Methods:

Specific aim 1: Evaluating the effect and prognosis of IMRT on the olfactory function in H & N cancer patients by using a smell identification tool designed for Taiwanese.

Patients

This study will enroll 100 patients initially diagnosed with H & N cancer at the Chiayi branch of Chang Gung Memorial Hospital in three years. The inclusion criteria include 1. patients should be older than 20 years old, 2. the H & N cancer treatments should contain IMRT, and 3. they should be willing to sign an informed consent approved by the institutional review board. The exclusion criteria include 1. patients with severe olfactory dysfunction (anosmia) and 2. patients with sinusitis before H & N cancer treatment.

# Note 1**:** we will apply TWSIT to screen patient's olfactory state and if the result is anosmia, the patient will be excluded.

# Note 2: If a patient is found to have sinusitis before treatment for H & N cancer, but if the sinusitis is completely resolved after adequate treatment before IMRT, the patient can still be included in this study.

Basic data

Before this trial, these enrolled patients will be recorded: 1. age and gender, 2. TNM, stage and images of their H & N cancer, planned dose and field of IMRT, 3. if having a habit of smoking, 4. if having the following medical comorbidities, including diabetes mellitus, hypertension, heart disease, chronic kidney disease, autoimmune disease, liver cirrhosis, chronic obstructive pulmonary disease, and 5. whether having received nasal turbinate, septum or sinus surgery.

Time points of evaluation

The time points for olfactory-associated assessments in the study include 1. before IMRT, 2. at the time of IMRT completed, 3. 1, 3, 6, and 12 months after IMRT. At each time points, the results of examination will be labelled as “primary assessment”. However, if sinusitis is noted at the primary assessment, adequate treatment will be given, and an intact evaluation will be repeated if the sinusitis is completed resolved. The results of repeated evaluation will be labelled as “secondary assessment”.

Evaluation content

The olfactory-associated assessments applied at each time points include 1. Taiwan version of smell identification test (TWSIT), 2. Sino-nasal Outcome Test -22 (SNOT-22), 3. nasal endoscopy, 4. collection of nasal secretions for cytokine analysis. The following describes the details of these detection methods in order.

Tool for testing smell function: TWSIT

Shen et al conducted a market survey and identified eight odorants, including 1. honey peach, 2. passion fruit, 3. cantaloupe, 4. lemon, 5. smoked plum, 6. garlic, 7. coffee, and 8. jasmine with a high identification rate (more than 95%) for 1000 Taiwanese. Then, based on the 8 odors, they developed a tool for smell identification specific for Taiwanese, the TWSIT

questionnaire consisted of multiple-choice questions. Correlational analysis was done for TWSIT and TC-UPSIT and the Spearman’s rho correlation coefficient between the two methods was 0.874 ($p < 0.01$), which indicated the newly developed tool had well validation with TC-UPSIT

Testing method of TWSIT

The device uses a sticker containing special odor molecules to determine whether the patient's sense of smell is lost and its severity by identifying the type of odor. The process of using this tool is described as follows:

1. The device contains a total of 16 questions, the first 8 questions are the same as the last 8 questions, but the order is different. Each question contains 2 sub-questions (Zone A and B). A total of 32 columns should be filled in and one is indispensable.
2. The patient uses a pencil to draw the olfactory block in the test book. This action will release the odor molecules contained in this block.
3. The patient approaches this block for about 1 cm with his or her nose and smells the smell of this block to answer the questions about the smell.
4. Each question contains two parts that need to be answered: Zone A and B. In Zone A, the patient needs to answer what he or she smells (there are four options for the question and cannot be blank). In Zone B, the patient must answer that the answer just now is 1. guess, 2. smell but not sure, 3. did smell, and he or she must choose one of the three, not blank.
5. After answering questions 1 to 8, the patient should take a rest for at least 1 minute. During the break, he or she should open the back page of page 8 and use various angles of the pencil to fill the box to remove the odor molecules attached to the pencil, which can avoid affecting the answering of subsequent questions. After waiting for 1 minute, the patient should continue to answer the 9 to 16 questions.
6. After completing the 16 answers, the patient hands over the testing device to a research-assistant for scoring.

Note 1: If the patient smells any smell, but cannot find the corresponding answer in the options, choose a similar smell to answer. In this case, "*smell but not sure*" should be marked in Area B, and "guess" should not be marked.

Note 2: There is only one situation in which the patient can choose the "guess", and that is when he or she can't smell anything at all.

Scoring method

The scoring method of the olfactory testing device is explained as follows:

1. Zone A: 1 point for correct, 0 point for wrong.
2. Zone B: 0 point for "guess", 1 point for "smell but not sure", 2 points for "did smell".

Note 1: Zone A fills in error and zone B fills in "smell but not sure" to get 1 point.

Note 2: Zone A fills in error and zone B fills in "did smell" to get 0 point.

The testing book has a total of 16 questions; the maximum score for the zone A is 16 points, and the maximum for the zone B is 32 points. Summing the scores of zone A and B gets a full score of 48 points. According to the score, it can be judged that the sense of smell is *normal, hyposmia, or anosmia*.

Age / Olfactory status	**Anosmia**	**Hyposmia**	**Normal**
20-35 years old	0-11 points	12-43 points	44-48 points
36-55 years old	0-11 points	12-42 points	44-48 points
56-80 years old	0-11 points	12-39 points	40-48 points

Note: The results of examinations under 19 years of age are interpreted according to the standard of 20-35 years; results of persons over 81 years of age are interpreted according to the standard of 56-80 years old.

SNOT-22

SNOT-22 is a subjective assessment tool commonly used to assess the severity of sinusitis.[14,15] At each time point of smell assessment, we will apply the tool to evaluate the subjective symptoms of sinusitis in those NPC patients.

There are 22 questions in the tool and each question is divided into 1. no problem, 2. very mild problem, 3. moderate problem, 4. severe problem, and 5. problem as bad as it can be. According to the severity, each problem corresponds to 0 to 5 points. After the patient has checked the severity of each question, all the scores are summed up.

Endoscopic evaluation

Because patients with H & N cancer received IMRT, there is a high incidence of causing sinusitis.[12] Further, sinusitis generally also affects olfactory function,[11] which will lead to errors in olfactory evaluation. Therefore, at each time point of the olfactory evaluation, we perform nasoendoscopy (OLYMPUS EVIS EXERA II CLV-180; OLYMPUS EVIS EXERA II CV-180; OLYMPUS ENF-VQ / VT2) to investigate whether sinusitis occurs; besides, we also observe the state of olfactory cleft to see whether the space is obliterated by the edematous nasal mucosa.

The key parts of endoscopic evaluation and recording include: 1. inferior turbinate, 2. middle meatus, 3. olfactory cleft, and 4. nasopharynx. In addition, if sinusitis is found, appropriate treatment will be given. During the follow-up period, the endoscopy will be repeated to evaluate whether the sinusitis is completely resolved, and a second smell-associated assessment will be made.

Modified Lund Kennedy endoscopic system

The modified Lund Kennedy (MLK) system is a common scoring tool to evaluate and quantify the severity of sinusitis. Therefore, we will use the scoring tool for each endoscopic evaluation. The MLK system is divided into three assessment aspects: 1. nasal polyps: “no” for 0 point, “limited in the middle meatus” for 1 point, and “exceeding the middle meatus” for 2 points; 2. edema: “no” for 0 point, “slight” for 1 point, “severe” for 2 points; 3. secretion: “no” for 0 point, clear secretion for 1 point and thick or purulent discharge for 2 points. After the nasal cavity on both sides are examined separately, the scores are summed. The maximum score on one side is 6 points; the maximum score on both sides is 12 points.[16]

Acoustic rhinometry

The olfactory epithelium is above the space of olfactory cleft. In addition to using nasoendoscopy to directly observe the changes in the state of this area during IMRT, we also apply acoustic rhinometry to subjectively evaluate the nasal resistance and volume for this region.

The examination can generate three sets of data (A. nasal resistance, NR and B. minimal cross- area, MCA) for 1.the nasal valve, 2.the middle part of nasal cavity, including the middle meatus and olfactory cleft, and 3. nasopharynx.

Measurement process

The patient adopts a standing posture, and the research assistant first makes a validation for the device (5 seconds), and then measures the nasal cavities on both sides. During the detection process, the patient needs to hold his or her breath for about 5-10 seconds in accordance with the instructions of the assistant. The assistant records the data of nasal resistance and cross-sectional area of the patient's bilateral nasal cavity for subsequent analysis.

Specific aim 2: Investigating the association between the IMRT-related olfactory dysfunction and nasal microenvironment

We collect the patient's nasal secretions and analyze the levels of cytokines at the time points when the patient was accessed with olfactory-related tests. Further, investigations will be conducted to analyze if it exists any correlation between the changes in the nasal microenvironment and IMRT-related olfactory dysfunction.

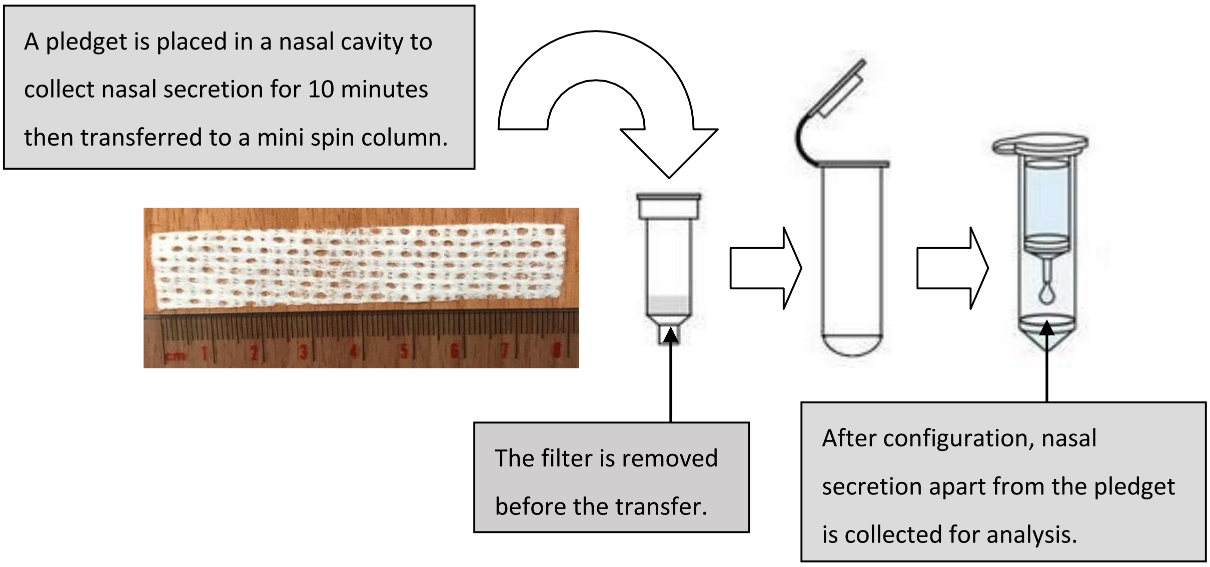

Sample collection

A pledget (8x2x0.2cm) will be placed gently into the anterior nasal cavity of patients for 10 minutes. After removal of the pledget containing nasal secretion, it will be put into the inner tube of a mini spin column (Epoch Life Science Inc.), which filter is removed in the preceding procedure. Centrifugation with 10000 bpm for 10 minutes will be performed and we will collect the nasal secretion separated from the pledget for further analysis and the samples will be stored at -80°C.

Sample analysis - Flow cytometry with cytometric bead array (CBA)

Soluble Th1-related cytokines including IL-1 beta, TNF-alpha, IFN-gamma and Th2-related cytokines, including IL-4, IL-6 and IL-10 will be measured by multiplexed cytometric bead array (CBA; BD Biosciences, San Jose, CA, USA). The CBA will be conducted according to the

manufacturer's instructions. A master cocktail containing the proteins of interest (as supplied by BD Biosciences) at a concentration of 5000pg/mL in CBA assay diluent will be prepared. 10 standard solutions of the following concentrations will be then prepared using the standard diluent (BD Biosciences): 5000 pgmL^{-1}, 2500 pgmL^{-1}, 1250 pgmL^{-1}, 625 pgmL^{-1}, 312.5 pgmL^{-1}, 158.25 pgmL^{-1}, 78.125 pgmL^{-1}, 39.0625 pgmL^{-1}, 19.53125 pgmL^{-1} and 0 pgmL^{-1}. The capture beads supplied with the kit for the proteins of interest will be vortexed for 15 seconds and diluted 1:100 in bead diluent to make up the bead cocktail. 25µL of the bead cocktail will be added to 25µL of samples/standards in a 96-well plate. The plate will be mixed on a shaker at 500rpm for 5 minutes before incubating for 1 hour at room temperature. The detector cocktail will be prepared in the same way as the bead cocktail, substituting the capture beads for detector antibodies (as per the BD manufacturer's protocol) and the bead diluent for detector diluent. 25µL of the detector cocktail will be then added to the wells and the plate mixed as before and left to incubate at room temperature for 2 hours. After incubation will be completed, 200µL of wash buffer (BD Biosciences) will be added to each well and the plate centrifuged at 1300rpm for 10 minutes at room temperature. The supernatant will be discarded, and the beads re-suspended in 50µL of wash buffer before being transferred into a tube for analysis. A BD FacsCanto II Flow Cytometer will be used to measure the fluorescence of the samples/standards. Using the FCAP Array software version 3.0.1 (BD Biosciences), the mean fluorescent intensities of the standards will be used to generate a standard curve for each protein of interest. The mean fluorescent intensities of the samples will be measured against the standard curve to give the concentrations of the proteins in pgmL^{-1}.

Statistical analysis

The sample size was based on a power of 80%. Using a two-tailed *t* test with $p < 0.05$, we

estimated the sample size as 60 participants, with an allowance for a potential dropout rate of 20%. The demographic characteristic and comorbidities of those H & N cancer cases are described as mean with standard deviation. Student *t* test will be used to analyze the variation of TW-SIT scores and the results would be plotted as multiple sloping chart. All data were analyzed using Microsoft Excel and MedCalc statistical software. A p value of <0.05 was considered statistically significant.

Anticipated Results:

Timetable of this project is listed following:

The first year

1. Recruit 40 newly diagnosed H & N cancer patients and complete evaluation, including TW-SIT, SNOT-22, endoscopy, acoustic rhinometry and collection of nasal secretion.
2. Make the preliminary analysis, including the alternative trend of olfactory function before and after IMRT and the correlation between the changes of smell and nasal microenvironment.
3. Write the interim report.

The second year

1. Recruit another 40 newly diagnosed H & N cancer patients, complete evaluation and make the analysis.
2. Write the interim report.

The third year

1. Recruit another 20 newly diagnosed NPC patients, complete evaluation and complete the analysis.
2. Write the final report.
3. Submit the manuscripts to SCI journals.

Expected training for participating staff:

1. PIs and research assistants can learn how to administer a project to study the effect of IMRT for H & N cancer patient on their olfactory function; They can have a deep understanding of the practice, know the problems that would be encountered and need to be overcome during the research, and accumulate relevant experience.
2. The research assistant can be familiar with how to use the relevant tools, including TW-SIT, SNOT-22 and acoustic rhinometry. Otolaryngologists can also be familiar with the process of collecting nasal secretions for analysis.
3. Research assistants can be familiar with how to operate flow cytometry and CBA to test cytokines in nasal secretions.
4. PIs and research assistants can learn how to organize these olfactory-related data, make

appropriate statistical analysis and plot charts.

5. PIs can learn how to write and publish articles on this topic.
