# Supplementary material for: Radiotherapy and Smell Function in Head and Neck Cancer: A Nonrandomized Clinical Trial
Source: JAMA Netw Open. 2025 Dec 18;8(12):e2548547. doi: 10.1001/jamanetworkopen.2025.48547 (PMC12715645; doi:10.1001/jamanetworkopen.2025.48547)
Supplement: Supplement 2. — eFigure 1. Dose Distribution to the Olfactory Regions During Radiotherapy eFigure 2. The Results of Hosmer-Lemeshow Goodness-of-Fit Test eFigure 3. Correlation Between Radiotherapy Dose and the Reduction in Olfactory Scores From Before Radiotherapy to Within 1 Year After Radiotherapy eFigure 4. Receiver Operating Characteristic Curve for Estimating Olfactory Dysfunction Within 1 Year After Radiotherapy Based on Radiotherapy Dose eFigure 5. Changes in Olfactory Scores Over Time in Patients Receiving High-Dose Radiotherapy [file jamanetwopen-e2548547-s002.pdf]

Supplemental Online Content

Chang GH, Tsai YT, Lin MH, et al. Radiotherapy and smell function in head and neck cancer: a nonrandomized clinical trial. *JAMA Netw Open*. 2025;8(12):e2548547. doi:10.1001/jamanetworkopen.2025.48547

eFigure 1. Dose Distribution to the Olfactory Regions During Radiotherapy
eFigure 2. The Results of Hosmer-Lemeshow Goodness-of-Fit Test
eFigure 3. Correlation Between Radiotherapy Dose and the Reduction in Olfactory Scores From Before Radiotherapy to Within 1 Year After Radiotherapy
eFigure 4. Receiver Operating Characteristic Curve for Estimating Olfactory Dysfunction Within 1 Year After Radiotherapy Based on Radiotherapy Dose
eFigure 5. Changes in Olfactory Scores Over Time in Patients Receiving High-Dose Radiotherapy

This supplemental material has been provided by the authors to give readers additional information about their work.

© 2025 Chang GH et al. *JAMA Network Open*.

eFigure 1. Dose Distribution to the Olfactory Regions During Radiotherapy

Axial (A), coronal (B), and sagittal (C) views illustrate the radiotherapy dose distribution to the olfactory regions, delineated based on CT imaging. The olfactory bulb and fossa were identified using anatomical landmarks: the anterior boundary at the frontal sinus-cribriform plate junction and the posterior boundary at the planum sphenoidale. Isodose lines (1000–6000 cGy) indicate dose distribution, highlighting the mean doses received by the olfactory bulbs for correlation with olfactory function changes.

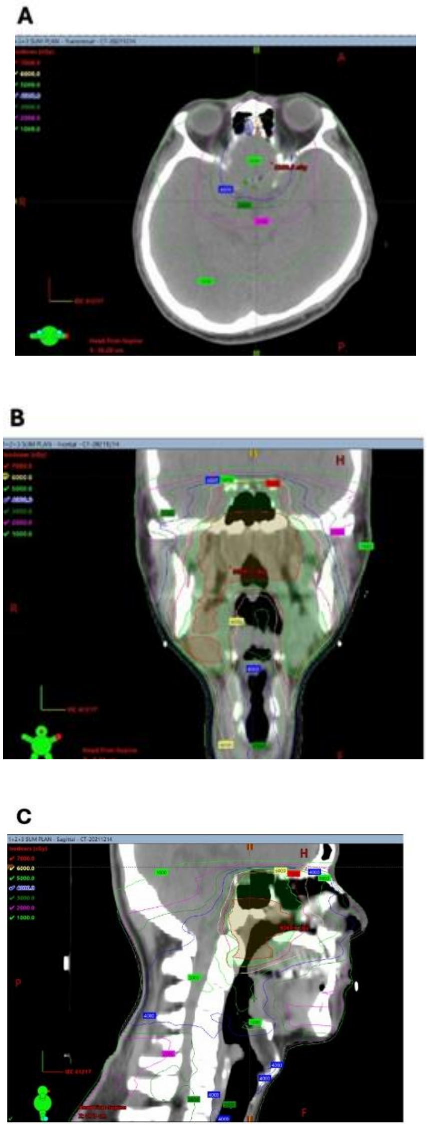

© 2025 Chang GH et al. *JAMA Network Open*.

eFigure 2. The Results of Hosmer-Lemeshow Goodness-of-Fit Test. There is no evidence of a lack of fit in the adjusted logistic regression model (p = 0.149). The test is used to indicate a good fitting model when its value is greater than 0.05 since it fails to reject the null hypothesis (there is no difference between the observed and model-predicted values), implying that the model's estimates fit the data at an acceptable level.

Partition for the Hosmer and Lemeshow Test

Group	Total	status = 1		status = 0	
		Observed	Expected	Observed	Expected
1	7	1	0.14	6	6.86
2	7	0	0.26	7	6.74
3	7	0	0.38	7	6.62
4	6	0	0.42	6	5.58
5	7	0	0.65	7	6.35
6	8	0	1.15	8	6.85
7	7	1	1.05	6	5.95
8	7	4	2.28	3	4.72
9	7	5	4.68	2	2.32

Hosmer and Lemeshow Goodness-of-Fit Test

Chi-Square	DF	Pr > ChiSq
10.7696	7	0.1490

© 2025 Chang GH et al. *JAMA Network Open*.

eFigure 3. Correlation Between Radiotherapy Dose and the Reduction in Olfactory Scores From Before Radiotherapy to Within 1 Year After Radiotherapy

This scatter plot illustrates the relationship between radiotherapy dose (Gy) and the reduction in olfactory scores (TWSIT). Each dot represents an individual patient's data. The regression analysis revealed an intercept of 1.226 and a slope of 0.128, with an R^2 value of 0.346, indicating a moderate positive correlation.

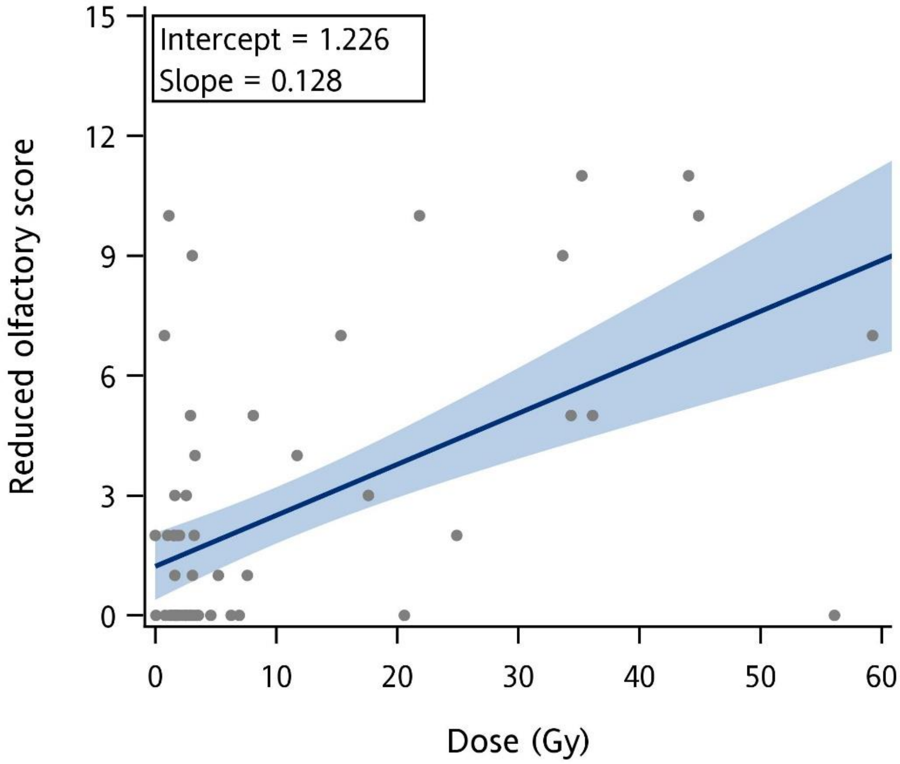

© 2025 Chang GH et al. *JAMA Network Open*.

eFigure 4. Receiver Operating Characteristic Curve for Estimating Olfactory Dysfunction Within 1 Year After Radiotherapy Based on Radiotherapy Dose

The receiver operating characteristic (ROC) curve illustrates the relationship between true positive and false positive rates in predicting olfactory dysfunction within one-year post-radiotherapy. An AUC of 0.74 indicates a moderate correlation between radiotherapy dose and the likelihood of olfactory dysfunction.

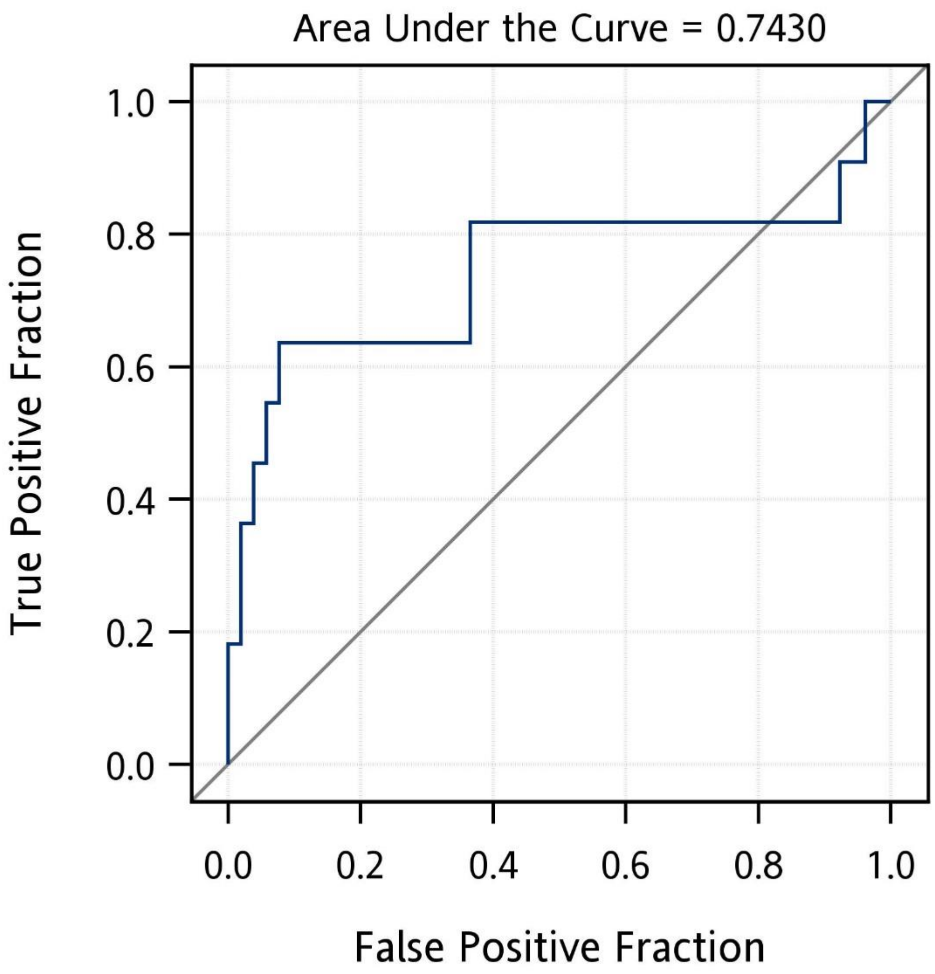

© 2025 Chang GH et al. *JAMA Network Open*.

eFigure 5. Changes in Olfactory Scores Over Time in Patients Receiving High-Dose Radiotherapy

This box plot illustrates the distribution of TWSIT olfactory scores in patients exposed to radiotherapy doses above 2200 cGy at different time points: pre-radiotherapy (Pre-RT), immediately post-radiotherapy (End-RT), and 1, 3, 6, and 12 months after treatment. The mean score (top of the box) showed a slight decline post-radiotherapy, with gradual stabilization over time, although variability increased (whisker: standard deviation).

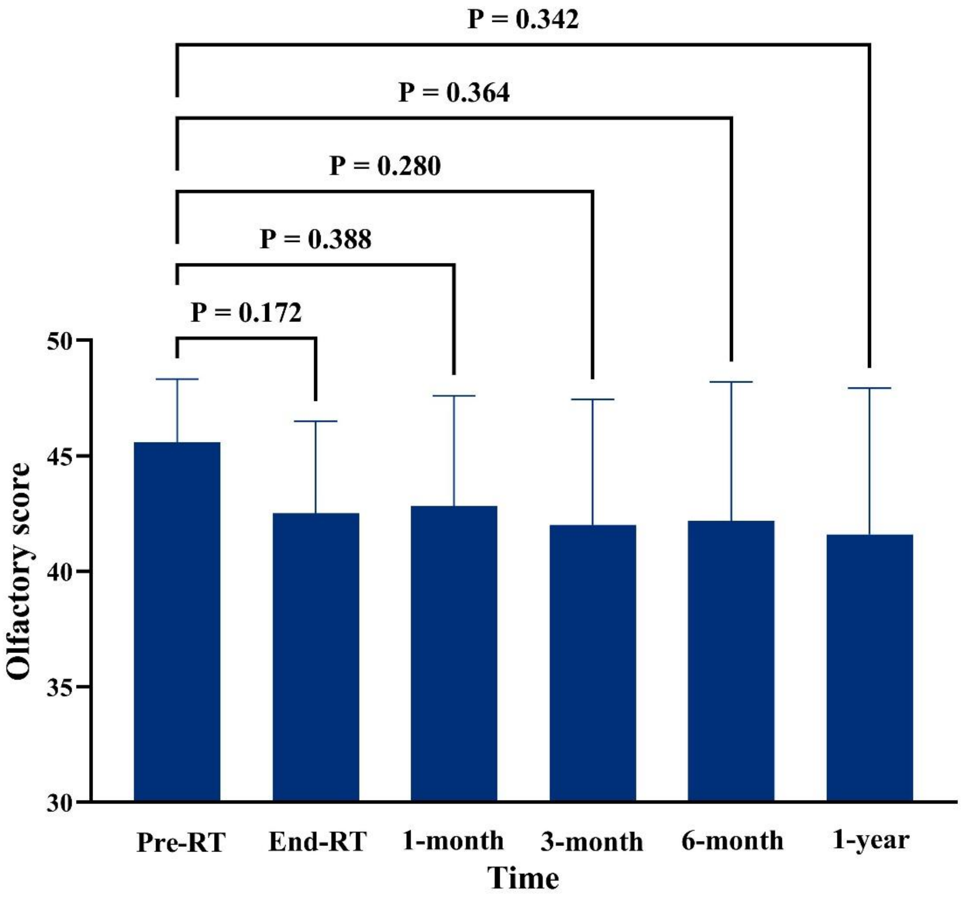

© 2025 Chang GH et al. *JAMA Network Open*.
