# Supplementary material for: Radiotherapy and Smell Function in Head and Neck Cancer: A Nonrandomized Clinical Trial
Source: JAMA Netw Open. 2025 Dec 18;8(12):e2548547. doi: 10.1001/jamanetworkopen.2025.48547 (PMC12715645; doi:10.1001/jamanetworkopen.2025.48547)
Supplement: Supplement 3. — Data Sharing Statement [file jamanetwopen-e2548547-s003.pdf]

Data Sharing Statement

Data
Additional Information: ISRCTN; https://www.isrctn.com/ISRCTN14947812; ISRCTN14947812
Data available: Yes
Data types: Deidentified participant data
How to access data: danielchen@adm.cgmh.org.tw
When available: With publication

Supporting Documents
Document types: None

Additional Information
Who can access the data: Researchers whose proposed use of the data has been approved
Types of analyses: DeIdentified data
Mechanisms of data availability: after approval of a proposal,
